# Extractive Fermentation for Process integration and amplified pullulan production by *A*. *pullulans* in Aqueous Two Phase Systems

**DOI:** 10.1038/s41598-018-37314-y

**Published:** 2019-01-10

**Authors:** Parul Badhwar, Punit Kumar, Kashyap Kumar Dubey

**Affiliations:** 1 Microbial Process Development Laboratory University Institute of Engineering and Technology Maharishi Dayanand University, Rohtak, 124001 Haryana India; 2 Bioprocess Engineering Laboratory, Department of Biotechnology Central University of Haryana, Mahendergarh, 123031 Haryana India

## Abstract

Extractive fermentation technique or *in situ* product recovery process is a novel technique to segregate the desired product simultaneously in a fermentation process. For economic and high yield production of pullulan, Extractive fermentation process was applied fermentation process of *A*. *pullulans*. Aqueous Two Phase system (ATPS) systems were designed with various molecular mass of PEG (400, 600, 4000 and 6000) and dextran or mono/bi-sodium phosphate salts. Systems with short Tie Line length (TLL) 6.7 and 7.5% w/w for PEG-Salt and PEG-dextran respectively were chosen. Volume ratio for all the systems was kept constant at 1.0 and pH 7.0 for PEG-dextran and PEG-NaH_2_PO_4_ was maintained, whereas pH 9.0 was kept for PEG-Na_2_HPO_4_. *A*. *pullulans*, was found to be viable with PEG-NaH_2_PO_4_ and PEG-dextran systems. The biomass partitioned in the PEG rich top phase and the exopolysaccharide pullulan shown affinity towards the bottom phase. A maximum yield (36.47 g/L) was found with PEG 4000-Dextran 500 system of extractive fermentation process. The proposed process aptly integrates upstream and downstream process for continuous production and recovery of pullulan from the biomass, thus reducing the time quotient of the whole process.

## Introduction

The development and designing of an efficient bioprocess is the most crucial step in any biotechnology industry. Every industry be it food, pharmaceutical, cosmetology or any other, wishes to design an economical and robust process that can be reproducible effortlessly. The increasing need to bring down the elevated product cost ultimately deduces on process development. A sequential array of multiple unit operations under upstream and downstream process forms a bioprocess. The forthcoming tendency in drafting an economical bioprocess is integration of several unit operations in a single step. To decrease or integrate a number of unit operations involved, it is highly required to design a distinct processing stage that accomplishes the objectives of the unit operations being replaced. ATPS has got wide practice in extraction, separation, purification, and upgradation of biomolecules, as well as in separating precious metals, sewage water treatment and detection of drug residues in food samples. ATPS is a liquid-liquid fractionation technique, based on the simple phenomenon of immiscibility of two aqueous solutions such as polymer-polymer (e.g. polyethylene glycol-PEG and dextran) and polymer-salt (e.g. phosphate, citrate, sulphate)^1,2^. Other than conventional polymer and salts, various other components of aqueous systems include smart polymers^3^, poly-propylene glycol-PPG^4^, polyethylene oxide sulphide-PEOS^5^, alcohols^6^, organic acids^7^, surfactants^8^, as well as ionic liquids^9^. Aqueous two phase separation techniques and its plausible variants possess the required characteristic for process integration. Extractive fermentation supports rapids exclusion of product into separate phase, thus circumventing product inhibition and degradation in a bioprocess. The method have been validated with synthesis and retrieval of cyclodextrins (CDs) from *Bacillus cereus* cyclodextrin glycosyltransferase^10^, metallic single walled carbon nanotubes (SWCNTs) from semiconducting SWCNTs has also been reported^11^. Fermentative production and separation of ethanol as well as acetone-butanol using simple sugar (glucose) as substrate by *Saccharomyces cerevisiae and Clostridium acetobutylicum* respectively was done with PEG-dextran as the phase forming components^12^.

The three major areas of research where ATPS proves a better alternative are extractive bioconversion, extractive fermentation, and integration of primary downstream process such as cell disruption and purification^13^. Albertson (1970), laid the foundation behind the concept of partitioning of cells and macromolecules in ATPS. The research was further extended to separate cells according to their surface properties with high selectivity and resolution^14,15^. The prime objective of studying ATPS here is to replace and integrate various unit operations involved in fermentative production of pullulan, with the single stage aqueous partitioning. Extractive fermentation or *in situ* product removal, is a novel process deriving from ATPS where the microbial system grows in one phase and the product of interest being extracellular in nature is excreted out and partitioned towards the other phase. Consequently the product of interest is extracted without disturbing biomass.

**Figure 1:**
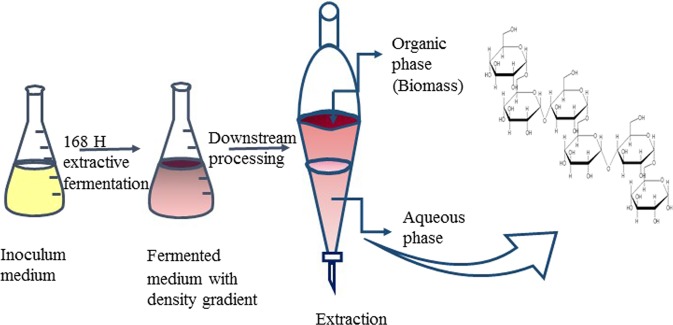
A symbolic flow diagram for the process integration of pullulan production with extractive aqueous two phase fermentation process.

*A*. *pullulans* is a black yeast like fungus, acknowledged mostly for its capability of producing pullulan as extracellular polysaccharide. Pullulan is an extraordinary biopolymer with many structural and functional characteristic features, multidisciplinary applications and innumerable patents. The unique α (1→4) glycosidic linkage between maltotriose unit and α (1→6) glycosidic bond connecting consecutive maltotriose units, laud the polymer with exclusive physical properties such as adhesiveness, film and fiber forming capacity^16^. Pullulan has got extensive application in the field of food, pharma, cosmetics, clinical and healthcare industries along with other miscellaneous uses. Pullulan as intensifier and starch substituent has been used in sauce, syrups, pickle and preservative^17^. Pullulan films are clear, edible, moldable, homogeneous, heat sealable, printable, and impermeable to oxygen. Composite membrane of pullulan with gelatin, lipid, protein, rice wax and antibiotics have further increased the quality of the pullulan films^18^. Despite all the advantage and applications of pullulan, its high cost has restricted its use and commercialization on grand scale. The fermentative synthesis of pullulan has been extensively studied and well described. But the downstream processing of the exopolysaccharide follows the same conventional process of biomass removal, organic acid precipitation followed by drying to obtain pulverized pullulan. The whole process endure more economic and environmental stress due to the excessive use of organic solvents. It is also considered that downstream process contributes major factor for high cost of biomolecules^19^. The number of unit operations involved directly tolls upon the quality and yield of pullulan. A symbolic representation of the extractive fermentation process attempts to justify the logic (Fig. 1).

The use of ATPS in pullulan recovery and partial purification has been previously described, but extractive fermentation has not yet been employed for the exopolysaccharide production^20^. Till date, no literature in the author’s awareness have been testified that exploits the probable application of ATPS for the *in situ* retrieval of pullulan. In the present study, the viability of ATPS on growth and cultivation of *A*. *pullulans* to produce pullulan was assessed in biphasic systems. The effect of dextran, phosphate salt concentration and various PEG molecular mass upon microbial growth and pullulan yield was considered to select the appropriate ATPS system for *in situ* recovery of pullulan.

## Results

### Partition and growth of *A*. *pullulans* in polymer-salt system

A range of PEG molecular weight i.e. 400, 600, 4000, 6000 was chosen to study the partition behavior of *A*. *pullulans*. In all the eight plausible systems of Polymer (PEG) with mono- and bi- sodium phosphate salt, the biomass has shown preference for top phase (PEG). However, a slight shift in biomass appearance towards the interface was observed with higher molecular mass of PEG 6000 (Tables 1 and 2). Such a trend in biomass phase preference with respect to polymer molecular mass is already in agreement with the established literature.

**Table 1:** Biomass phase preference of *A*. *pullulans* in Aqueous Two Phase systems of PEG-mono sodium phosphate (TLL 6.7% w/w, V_R_ 1.0, pH 7 were kept constant in all the systems).

System	PEG molecular mass	PEG (% w/w)	Monosodium Phosphate (% w/w)	Biomass preference	Dry Cell weight (g/L)
1. PEG400-phosphate	400	6.13	18.77	Top phase	24.75 ± 0.91
2. PEG600-phosphate	600	5.4	23.42	Top phase	19.95 ± 1.33
3. PEG4000-phosphate	4000	6.71	19.6	Top phase	16.54 ± 1.02
4. PEG6000-pohsophate	6000	6.32	19.4	interphase	15.37 ± 0.50

**Table 2:** Biomass phase preference of *A*. *pullulans* in various in Aqueous Two Phase systems of PEG-bi-sodium phosphate (TLL 6.7% w/w, V_R_ 1.0, pH 9 were kept constant in all the systems).

System	PEG (g/mol)	PEG (% w/w)	Bisodium Phosphate (% w/w)	Biomass preference	Dry Cell weight (g/l)
5. PEG400-phosphate	400	6.13	7.37	Top phase	11.47 ± 0.50
6. PEG600-phosphate	600	5.4	6.2	Top phase	9.50 ± 0.53
7. PEG4000-phosphate	4000	6.71	6.64	Interphase	slight
8. PEG6000-phosphate	6000	6.32	6.2	Interphase	negligible

**Figure 2:**
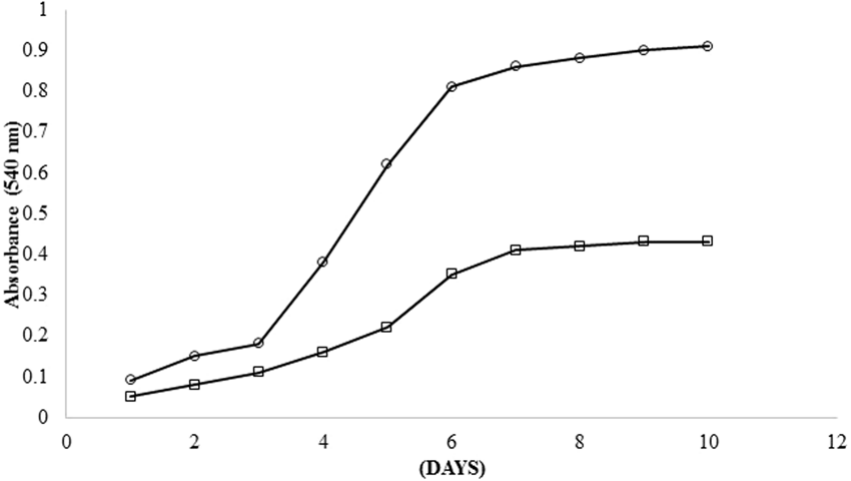
*A*. *pullulans* growth pattern on PEG-Monosodium phosphate salt (○) and PEG-Bisodium phosphate (□) aqueous two phase systems, the cell growth was determined measuring optical density at 540 nm. Both the systems were maintained at similar TLL 6.7% w/w, V_R_ 1.0 but varying pH 7 and 9 respectively.

The two pH values i.e. 7.0 and 9.0 (NaH_2_PO_4_/Na_2_HPO_4_) had direct influence on biomass viability (Fig. 2). A slight increment in pH had negligible effect on pullulan production but higher pH values resulted in decreased cell viability and thus reduced amount of pullulan produced^21^. To observe the sole effect of PEG upon biomass, the growth medium was exclusively incubated with PEG of different molecular masses (400, 600, 4000, and 6000). Biomass growth was observed in all the systems with varying molecular mass of PEG.

### Partition and growth of *A*. *pullulans* in polymer-polymer system

The partition behavior of *A*. *pullulans* was evaluated in PEG (400, 600, 4000, and 6000) and dextran T500. As observed earlier in polymer-salt systems, biomass here also partitioned in top phase i.e., PEG rich phase (Table 3). All the systems of PEG-dextran well supported the growth and viability of *A*. *pullulans*. In fact, the dry cell mass in PEG-Dextran systems was double fold in scale with respect to PEG 400-monosodium phosphate salt system (2.42 g/l, maximum DCW in PEG-salt systems, Tables 1–3).

**Table 3:** Biomass phase preference of *A*. *pullulans* in various in Aqueous Two Phase systems of PEG-dextran (TLL 7.5, V_R_ 1.0, pH 7 were kept constant in all the systems).

System	PEG (g/mol)	PEG (% w/w)	Dextran (% w/w)	Biomass preference	Dry Cell weight (g/l)
9. PEG400-Dextran T5OO	400	6.13	5.98	Top phase	29.31 ± 0.46
10. PEG600- Dextran T5OO	600	5.4	4.9	Top phase	37.62 ± 0.68
11. PEG4000- Dextran T5OO	4000	6.93	6.05	Top phase	45.44 ± 0.74
12. 12. PEG6000- DEXT5OO	6000	5.09	4.9	Interphase	34.39 ± 0.49

### Phase preference of pullulan and comparative yield

PEG-Dextran system and PEG-monosodium phosphate salt system supported the growth of *A*. *pullulans*. Thus, they were evaluated for the *in situ* recovery of pullulan and the results are presented in terms of partition coefficient (K_p_) values (Table 4). While the biomass preferred PEG rich top phase, pullulan partitioned in dextran/salt rich bottom phase. Polarity of the exopolysaccharide with respect to the phase component drives its movement towards dextran/salt rich phase. It was observed that the K_p_ value for all the systems was less than 1 (Tables 4 and 5). Among all the PEG-dextran systems, pullulan partition and recovery was best observed with higher molecular weight PEG i.e. PEG 6000 (Table 4, partition coefficient K_p_: 0.022; top phase recovery: 1.8; bottom phase recovery: 98.2).

**Table 4:** Pullulan phase preference and partition behavior in Aqueous Two Phase systems of PEG-mono sodium phosphate (TLL 6.7% w/w, V_R_ 1.0, pH 7 were kept constant in all the systems).

System	PEG (% w/w)	Monosodium Phosphate (% w/w)	Top phase recovery (%)	Bottom phase recovery (%)	Partition coefficient (K_P_)
1. PEG400-PHOSPHATE	6.13	18.77	8.2	91.8	0.094
2. PEG600-PHOSPHATE	5.4	23.42	4.6	95.4	0.049
3. PEG4000-PHOSPHATE	6.71	19.6	2.5	97.5	0.028
4. PEG6000-POHSOPHATE	6.32	19.4	1.9	98.1	0.022

**Table 5:** Pullulan phase preference and partition behavior in Aqueous Two Phase systems of PEG-dextran (TLL 7.5, V_R_ 1.0, pH 7.0 were kept constant in all the systems).

System	PEG (% w/w)	Dextran (% w/w)	Top phase recovery	Bottom phase recovery	Partition coefficient (K_P_)
5. PEG400-DEXT500	6.13	5.98	4.8	95.2	0.056
6. PEG600- DEXT500	5.4	4.9	4.1	95.9	0.048
7. PEG4000- DEXT500	6.93	6.05	2.1	97.9	0.026
8. PEG6000- DEXT500	5.09	4.9	1.8	98.2	0.019

**Figure 3:**
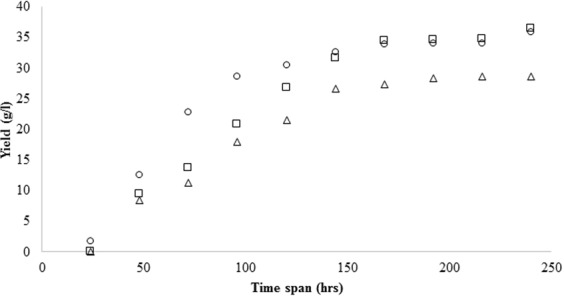
Yield of pullulan was observed in Aqueous two phase systems, PEG 6000-Dextran (□) and PEG-Monosodium phosphate salt (▵) and compared with standard pullulan yield (○) via conventional fermentation process in a span of 10 days. The culture conditions for fermentative pullulan production were 6.5 of ph at 200 rpm and 28 °C of temperature whereas for PEG-dextran 7.5% w/w TLL, VR 1.0 and 7.0 pH.

As PEG-dextran ATPS allows a higher recovery (≥95%) of pullulan with best results obtained for higher molecular weight PEG, that facilitates phase separation and product recovery. *in situ* recovery of pullulan was then compared with standard fermentation procedure (Fig. 3). Pullulan yield in PEG 6000-dextran and PEG 6000-monosodium phosphate system was evaluated after each 24 hours of fermentation.

### Pullulan characterization

**Figure 4:**
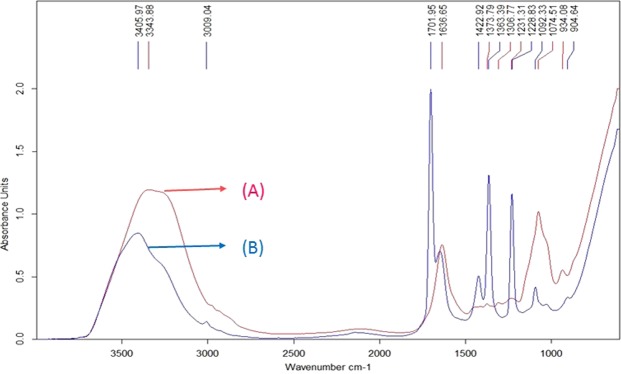
The FTIR absorption spectrum of standard Pullulan (**A**) and pullulan sample (**B**) obtained from extractive fermentation systems of ATPS, for characterization and identification of major bonds and configuration.

The FT-IR spectra of commercial pullulan used as standard and those of pullulan sample obtained in extractive fermentation procedure were evaluated and compared in Fig. 4. Alpha configuration considered as the distinguishing feature of pullulan exists in the absorption range of 650–1000 cm^−1^. Absorption peaks at 686.81 cm^−1^ and 936.28 cm^−1^ for standard pullulan, and pullulan sample respectively was observed. Peaks in the range of 900 and 650 cm^−1^ proves the presence of α-1, 6 and α-D-glucopiranosid units, respectively (Fig. 4). Pullulan purity was also analyzed and was found that, the purity of pullulan was 56.89%.

### Commercial prospect of extractive fermentation process

For a process to be commercially valuable, it has to produce consistent results with ease of operation, magnification and replication. In scaling up a process to industrial level or to an extent of merchandizing, substance investment is one crucial factor. Thus for production of 1 kg of pullulan through the 3 processes (i.e. organic solvent precipitation (OSPPT), ATPS and its extension extractive fermentation) were also scrutinized in (Table 6). To produce a kg of pullulan, organic solvent thus precipitation method requires 28.57 L of fermented medium, which was slightly higher than extractive fermentation process (27.77 L medium) and ATPS process (23.80 L medium). The amount of chemical required in inoculum and fermentation medium changes accordingly. Other than the customary medium formulating reagents, ethanol, PEG, dextran and acetone were the process defined reagents. These four reagents essentially defines the process tariff. For organic solvent precipitation process double the volume of ethanol is required to precipitate out the biopolymer. 57.14 L of ethanol (priced $1608.71) was required to obtain 1 Kg of pullulan. Whereas PEG ($ 46.57 in extractive fermentation process), dextran ($ 7.33) and acetone ($ 215.15) came at very minimal price in comparison to ethanol, thus decreased the total process cost to several folds.

**Table 6:** Comparative analysis of capital investment between the proposed method i.e. Extractive fermentation, ATPS and Conventional organic solvent precipitation for the production of 1 kg of pullulan.

S.no	Process	Chemical requirements	OSPPT ($)	ATPS ($)	Extractive fermentation ($)
1.	Seed Culture	Sucrose	12.76	10.66	12.40
		Magnesium Sulphate	0.0039	0.0033	0.0038
		Sodium Chloride	0.041	0.034	0.040
		Ferrous Sulphate	0.0021	0.0018	0.0021
		Manganese Sulphate	0.0027	0.0022	0.0026
		Zinc Sulphate	0.0014	0.0012	0.0014
		Dipotassium Hydrogen Phosphate	1.13	0.94	1.10
		Ammonium Sulphate	0.16	0.13	0.15
2.	Fermentation	Sucrose	127.92	106.39	124.34
		Yeast	17.68	14.73	17.19
		Sodium Chloride	1.23	1.03	1.20
		Dipotassium Hydrogen Phosphate	28.24	23.53	27.45
		Ammonium Sulphate	1.56	1.30	1.51
		PEG	—	—	46.57
		Dextran	—	—	7.33
3.	Downstream processing	Ethanol	—	—	—
		Poly Ethylene Glycol	—	39.85	—
		Dextran	—	6.28	—
		Acetone	—	184.41	215.17
4.	TOTAL PRICE		**1799.44**	**389.29**	**454.46**

## Discussion

*A*. *pullulans*, is a black yeast like fungus found generously in many ecological niches. Thus the fungus can survive in a wide range of environmental conditions. In the various ATPS systems *A*. *pullulans* also survived, the viability was observed in the form of dry cell mass collected after extractive fermentation process. The biomass was indeed lesser then regular fermentation conditions (58.72 g/l), of biomass was observed in all the systems formed even a scant amount of microbial growth was observed with high molecular weight PEG (PEG-6000) and high bi-sodium phosphate salt concentration (Table 1). These results established that the high salt concentration in PEG-bi-sodium phosphate extractive fermentation systems does not support proper cell growth. As PEG has got no negative effect on the growth of *A*. *pullulans* in the ATPS systems, it was recommended to observe polymer-polymer ATPS system for microbial growth and pullulan production. It has been also reported that dextran is not utilized by microorganism during growth^22^.

Among all the four PEG-dextran systems, system 11 with PEG 4000 and dextran facilitated maximum cell growth and clear, distinct phases. It is very much clear that, between polymer-salt and Polymer-polymer systems, PEG-Dextran system readily supports the growth of *A*. *pullulans*, and can easily be delivered in extractive fermentation of ATPS.

Benavides *et al*.^21^ explained an increase in excluded volume at both the phases with rise in polymer molecular mass, this in turn results in particle and biomass movement towards the interface^23^. The phenomenon precisely justified the biomass shifting towards interface with higher molecular mass PEG and high pH (9.0).The phenomenon of particle shifting towards interface due to excluded volumes occurs at higher molecular weight of PEG, which was also observed with PEG 6000-dextran.

The bottom phase preference of pullulan could be explained in terms of polarity. Dextran has numerous hydroxyl groups along the chain whereas PEG has got only two hydroxyl groups at the terminal of the molecule^24^. The presence of oxygen in pullulan entitle its affinity towards dextran, since it has got numerous hydroxyl groups^16^. This was validated by the partition coefficient values. Since, pullulan has a low molecular weight (<1000 g/mol), the effect of excluded volume on the PEG 6000-Dextran system is negligible.

A huge difference in the amount of exopolysaccharide produced under the two systems is observed, with a significant *in situ* recovery of pullulan in PEG 6000-dextran system as compared to PEG 6000-salt system. Remarkably, the *in situ* pullulan recovery in PEG 6000-dextran system is also superior to conventional organic solvent precipitation protocol. This outcome highlights the shortcomings in the conventional process and possible product loss during tedious downstream processing scheme.

The results obtained in this study, justified the proposed advantages of Aqueous Two Phase system (ATPS) for the extractive fermentation of pullulan from *A*. *pullulans*. The PEG-dextran ATPS system was found to be suitable for the growth of *A*. *pullulans* in top phase and partitioning of pullulan to the other phase. The yield of the extractive fermentation process was found to be 36.47 g/L, slightly higher than organic solvent precipitation process (35.84 g/L) and lesser than ATPS process (42.17 g/L). The proposed process aptly integrates upstream and downstream process for continuous production and recovery of pullulan from the biomass, thus reducing the time quotient of the whole process. ATPS proved to be the most economical process with an expenditure of $ 389.29, followed by extractive fermentation process ($ 4454.46) and organic solvent precipitation process with a huge investment of $ 1799,44. Extractive fermentation or *in situ* recovery of pullulan proved to be an environmental friendly, reliable and reproducible process. The techniques could be easily adopted in large scale fermentation processes as a beneficial means in process integration.

## Methods

### Microbial strain

*A*. *pullulans* was procured from MTCC Chandigarh. The micro-organism was maintained at YPD medium at 25 °C and sub-cultured every 3 weeks. For fermentative production of pullulan, *A*. *pullulans* was grown in YPD (1.5% w/v Yeast, 2% w/v peptone, 15.5% w/v dextrose) media at 28 °C and 200 rpm for 48 H as inoculum^25^. It was then sub-cultured to final production medium (sucrose, ammonium sulphate along with yeast extract, sodium chloride and dipotassium hydrogen phosphate) for 7 days, ph 6.5, at 200 rpm and 28 °C of temperature^26^. The growth in liquid culture was routinely examined by measuring the optical density at 540 nm. All the fermentation studies were carried out in 250 mL Erlenmeyer flasks with a 50 mL working volume.

### Growth and partition of *A*. *pullulans* in ATPS system

Polymer-salt system was prepared using polyethylene glycol (PEG, Merck life Science) with molecular masses of 400, 600, 4000, 6000 (50% w/w stock solution). For polymer-salt system PEG with mono- and bi- sodium phosphate was selected and PEG-dextran system was selected as the Polymer-Polymer system. ATPS systems of PEG-Salt (40% w/w stock solution) with short tie line length (TLL)-6.7 and volume ratio (V_R_)-1.0 were chosen in the present procedure. Orthophosphoric acid or sodium hydroxide was added, for fine adjustment of pH. The polymer-polymer system was made up of PEG (50% w/w stock solution) and Dextran (20% w/w stock solution). PEG-Dextran ATPS systems with short TLL (7.5) and V_R_ 1.0 were selected. Dilution with pullulan production media was done to achieve the final volume of the system. Similar to systematic fermentation procedure, ATPS cultures of 100 g total weight were inoculated with 10% w/w from the seed culture. The culture conditions were also kept similar, pH 7.0, constant shaking at 200 rpm, 28 °C temperature for 10 days. The growth was monitored over 10 days in both PEG-phosphate and PEG-dextran ATPS systems, by measuring optical density at 540 nm using spectrophotometer. The phase preference of *A*. *pullulans* was determined visually. All the experiments were performed in triplicate. Standard pullulan was obtained from Sigma-Aldrich (Mumbai, India). The purity of pullulan was analyzed by pullulanase bioassay^27^.

### Phase partition of pullulan

At the end of fermentation experiments, the phases were cautiously filter separated using a separating funnel. The cell free phases thus obtained were then centrifuged (1000 × g for 10 mins) to remove other undissolved suspended particles. To both the top and bottom phase samples, double volume of acetone was added and mixed to precipitate the pullulan content. The system was then kept overnight to let the precipitates settle down. The precipitates were weighed and analyzed using FTIR and TLC. The FTIR spectra of the samples were recorded in attenuated total reflection (ATR) mode using a FTIR spectrometer (Bruker) and scanned from 400 to 3500 cm^−1^. Each absorption spectrum represented an average of 10 consecutive scans. For TLC analysis the pullulan obtained from the extractive fermentation process, was subject to enzymatic hydrolysis by pullulanase. Pullulanase specifically digests the specific α (1→6) bonding present exclusively in pullulan. Thus the harvested EPS was treated with 0.1 U ml^−1^ pullulanase for 6 H at 45 °C.

The partition coefficient for the pullulan precipitates was also calculated. The partition coefficient (K_p_) of pullulan was calculated using the equation

$${{\rm{K}}}_{{\rm{P}}}={{\rm{C}}}_{{\rm{T}}}/{{\rm{C}}}_{{\rm{B}}},$$

where, C_T_ and C_B_ are concentrations of pullulan in the top phase and bottom phases, respectively. Yield of pullulan was obtained by the constant weight of the precipitated exopolysaccharide obtained on drying at 60 °C.

### Effect of molecular mass of peg and phosphate concentration on viability and growth of *A*. *pullulans*

The polymer-salt systems of ATPS in fermentation medium were prepared with combinations of PEG (molecular mass 400, 600, 4000, and 6000) and monobasic/dibasic sodium phosphate at 7.0 and 9.0 pH. Similarly the polymer-polymer systems in extractive fermentation medium were prepared with PEG (400, 600, 4000 and 6000) and dextran. All the extractive fermentation systems were inoculated with growth phase culture (10% v/v). The microbial viability was monitored visually over the span of 10 days to determine the maximum concentration of salt, PEG and dextran that *A*. *pullulans* could tolerate. The biomass was determined by increment in turbidity and appearance of pink pigment in each solution. Absence of microbial growth and viable conditions were recognized when neither turbidity nor pigmentation appear over the defined time span.

### Economic study of the experiment

The proposed method, i.e. extractive fermentation process for obtaining pullulan was also analyzed in terms of capital investment. The economic viability of the process was investigated in comparison to the established organic solvent precipitation method and aqueous two phase separation process. The investment in terms of time (hours) spent for the production of 1 Kg pullulan powder in these three processes were also analyzed. The prices of the reagents consumed in the three mentioned processes were obtained from HiMedia (Mumbai, India) and Sigma Aldrich (India). The cost of reagents was calculated in United States Dollar (USD) using conversion of Indian Rupee into USD considering 1 United States Dollar equals to 69.8 Indian Rupee.

## Supplementary information

Consumption of sucrose during extractive fermentation process

## References

[1] Iqbal M (2016). Aqueous two-phase system (ATPS): an overview and advances in its applications. Biol. Proced. Online.

[2] Hatti-Kaul, R. *Aqueous Two-Phase Systems*. 11, (Humana Press, 2000).

[3] Cunha, M. T., Aires-Barros, R. & Cabral, J. M. S. Extraction for rapid protein isolation. In: Kaul, R. & Mattiasson, B. (Eds), *Isolation and Purification of Proteins. Marcel Dekker*. New York, pp. 321–372 (2003).

[4] Salabat A, Abnosi MH, Bahar AR (2007). Amino acids partitioning in aqueous two-phase system of polypropylene glycol and magnesium sulfate. J. Chromatogr. B.

[5] Hernandez-Mireles T, Rito-Palomares M (2006). New aqueous two-phase systems based on poly(ethylene oxide sulfide) (PEOS) and potassium phosphate for the potential recovery of proteins. J. Chem. Technol. Biotechnol..

[6] Wang Y, Mao Y, Han J, Liu Y, Yan Y (2010). Liquid−Liquid Equilibrium of Potassium Phosphate/Potassium Citrate/Sodium Citrate + Ethanol Aqueous Two-Phase Systems at (298.15 and 313.15) K and Correlation. J. Chem. Eng. Data.

[7] Saravanan S, Rao JR, Nair BU, Ramasami T (2008). Aqueous two-phase poly(ethylene glycol)–poly(acrylic acid) system for protein partitioning: Influence of molecular weight, pH and temperature. Process Biochem..

[8] Liu Y, Wu Z, Zhang Y, Yuan H (2012). Partitioning of biomolecules in aqueous two-phase systems of polyethylene glycol and nonionic surfactant. Biochem. Eng. J..

[9] Passos H (2012). Characterization of aqueous biphasic systems composed of ionic liquids and a citrate-based biodegradable salt. Biochem. Eng. J..

[10] Lin YK (2015). Direct recovery of cyclodextringlycosyltransferase from Bacillus cereus using aqueous two-phase flotation. J. Biosci. Bioeng..

[11] Tang MSY (2014). The Removal of Metallic Single-Walled Carbon Nanotubes Using an Aqueous Two-Phase System. J. Nanosci. Nanotechnol..

[12] Mattiasson, B. *et al*. Solvent production by Clostridium acetobutylicum in aqueous two-phase systems. In Chibata, I., Fukui, S. & Wingard, Jr. L. B. (Eds) *Enzyme Engineering*, vol. 6. Plenum, New York, pp. 153–155 (1982).

[13] Rito-Palomares M (2004). Practical application of aqueous two-phase partition to process development for the recovery of biological products. J. Chromatogr. B.

[14] Albertsson PA (1970). Partition of cell particles and macromolecules in polymer two-phase systems. Adv. Protein Chem..

[15] Zimmermann, S. *et al*. Cell Separation in Aqueous Two-Phase Systems – Influence of Polymer Molecular Weight and Tie-Line Length on the Resolution of Five Model Cell Lines. 1–31 (2017). 10.1002/biot.201700250 29087627

[16] Leathers TD (2003). Biotechnological production and applications of pullulan. Appl Microbiol Biotechnol.

[17] Pınar, O. & Yangılar, F. Pullulan: Production and usage in food ındustry. **4**, 57–63 (2013).

[18] Ferreira ARV, Alves VD, Coelhoso IM (2016). Polysaccharide-Based Membranes in Food Packaging Applications. Membranes (Basel)..

[19] Stanbury, P. F. Whitaker, A. & Hall, S. J. *Principles of fermentation technology (2016)*.

[20] Badhwar, P., Kumar, P. & Dubey, K. K. Development of aqueous two-phase systems comprising poly ethylene glycol and dextran for purification of pullulan: Phase diagrams and fiscal analysis. *Eng*. *Life Sci*. 1–26, 10.1002/elsc.201700156 (2018). 10.1002/elsc.201700156 PMC6999398 32624933

[21] Benavides J, Aguilar O, Lapizco-Encinas BH, Rito-Palomares M (2008). Extraction and Purification of Bioproducts and Nanoparticles using Aqueous Two-Phase Systems Strategies. Chem. Eng. Technol..

[22] Ghosh S, Swaminathan T (2003). Optimization of Process Variables for the Extractive Fermentation of 2, 3-Butanediol by Klebsiella oxytoca in Aqueous Two-phase System Using Response Surface Methodology. Chem. Biochem. Eng.

[23] Lacroix C, LeDuy A, Noel G, Choplin L (1985). Effect of pH on the batch fermentation of pullulan from sucrose medium. Biotechnol. Bioeng..

[24] Farris, S., Unalan, I. U., Introzzi, L., Fuentes-Alventosa, J. M. & Cozzolino, C. A. Pullulan-based films and coatings for food packaging: Present applications, emerging opportunities, and future challenges. *J*. *Appl*. *Polym*. *Sci*. **131**, n/a-n/a (2014).

[25] Choudhury AR, Bhattacharjee P, Prasad GS (2013). Development of Suitable Solvent System for Downstream Processing of Biopolymer Pullulan Using Response Surface Methodology. Plos One.

[26] Singh RS, Singh H, Saini GK (2009). Response Surface Optimization of the Critical Medium Components for Pullulan Production by *Aureobasidium pullulans* FB-1. Appl. Biochem. Biotechnol..

[27] Wu S-J, Kim J-M, Zhou C, Jin Z-Y, Tong Q-Y (2010). Estimation of pullulan by hydrolysis with pullulanase. Biotechnol. Lett..

