# Supplementary material for: Extractive Fermentation for Process integration and amplified pullulan production by A. pullulans in Aqueous Two Phase Systems
Source: Sci Rep. 2019 Jan 10;9:32. doi: 10.1038/s41598-018-37314-y (PMC6328562; doi:10.1038/s41598-018-37314-y)
Supplement: Supplementary file 1 — Consumption of sucrose during extractive fermentation process [file 41598_2018_37314_MOESM1_ESM.pdf]

# **Extractive Fermentation for Process integration and amplified pullulan production by *A. pullulans* in Aqueous Two Phase Systems**

Parul Badhwar[1], Punit Kumar[1], Kashyap Kumar Dubey [2,*]

[1]*Microbial Process Development Laboratory*

*University Institute of Engineering and Technology*

*Maharishi Dayanand University, Rohtak-124001*

*Haryana, INDIA*

[2]*Bioprocess Engineering Laboratory, Department of Biotechnology*

*Central University of Haryana, Mahendergarh-123031*

*Haryana-INDIA*

*Corresponding Author: Kashyap Kumar Dubey, Department of Biotechnology, Central University of Haryana, Mahendergarh-123031, Haryana-INDIA.

E-mail: kashyapdubey@gmail.com

SUPPLEMENTARY DATA

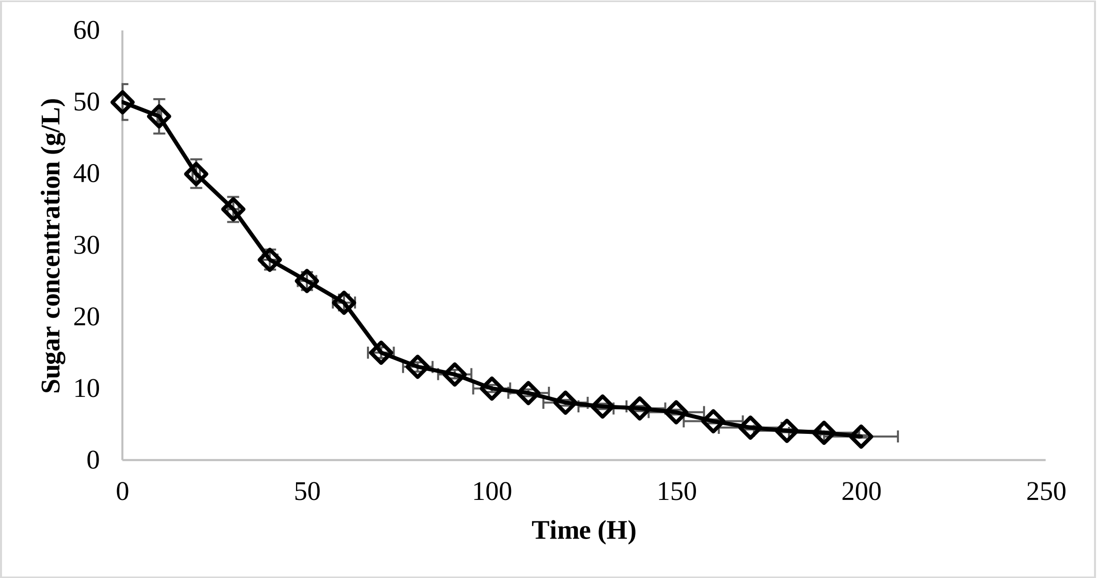


Figure: Consumption of sucrose during extractive fermentation process of *A. pullulans* for *in-situ* retrieval of pullulan. The process was carried out for 200 hr.
